# Longitudinal dynamics of intestinal bacteria in the life cycle and their effects on growth and development of potato tuber moth

**DOI:** 10.3389/fmicb.2025.1542589

**Published:** 2025-06-18

**Authors:** Qiaosi Fu, Wenqian Wang, Bin Chen, Yuxi Hu, Rui Ma, Enran Zhu, Sitong Jin, Haosheng Cai, Guanli Xiao, Guangzu Du

**Affiliations:** ^1^State Key Laboratory for Conservation and Utilization of Bio-Resources in Yunnan, College of Plant Protection, Yunnan Agricultural University, Kunming, China; ^2^College of Agriculture and Biology Technology, Yunnan Agricultural University, Kunming, China

**Keywords:** potato tuber moth, gut microbiota, 16S rRNA sequencing, different life stages, holometabolous development

## Abstract

Potato tuber moth (PTM), *Phthorimaea operculella* (Lepidoptera: Gelechiidae), is an oligophagous pest that damages potatoes. Intestinal microorganisms play important roles in regulating the life activities of host insects. The gut of PTM is rich in microbials, but it is unclear that the dynamics of the structure and diversity of intestinal bacteria in the different development period of potato tuber moth. In this study, the dynamics of the intestinal bacterial community across the whole life cycle of PTM were evaluated using single molecule real-time sequencing. The intestinal microbiota of PTM is predominantly composed of Proteobacteria and Firmicutes, and it is different with the difference of development stages. *Wolbachia endosymbionts* were the dominant species of intestinal symbiotic bacteria in eggs and the first-instar larvae. *Enterococcus mundtii* was the dominant species of intestinal symbiotic bacteria in the second, third, and the fourth instar larvae, as well as in both male and female pupae. Moreover, the predominant species of intestinal symbiotic bacteria in female adults is *Enterobacter ludwigii*, while the dominant bacterial species is *Serratia rubidaea* in male adults. Principal component analysis and non-metric Multi-dimensional scaling analysis confirmed the differences in intestinal symbiotic bacteria structure at different developmental stages. In addition, after reintroducing the bacteria following antibiotic treatment, it was found that the antibiotics significantly inhibited the development of the potato tuber moth, whereas the gut bacteria appeared to facilitate its growth. The findings of this study will enhance our understanding of intestinal microorganisms on the development of their host insects across the life cycle. Moreover, it will establish a foundation for elucidating the physiological functions of key microorganisms in the intestinal tract of the potato tuber moth, while also offering new insights and strategy to the biological control of this pest.

## 1 Introduction

The potato tuber moth (PTM), *Phthorimaea operculella* (Zeller) (Lepidoptera: Gelechiidae), which is widely distributed throughout the world (Rondon, 2010). The pest primarily inflicts damage on Solanaceae plants, especially on potato (Zheng et al., 2019). The PTMs possesses strong adaptability to the environment, remarkable reproductive capacity and parthenogenesis, thereby causing significant economic losses (Gao, 2018; Liu et al., 2018). Currently, PTM is prevalently present in potato-producing regions in southern China, particularly in areas such as Yunnan, Sichuan, and Guizhou, where the occurrence of this pest is highly severe. In recent years, as the planting area of China’s potato industry expands, the distribution area of this pest shows a tendency to continuously expand (Pan et al., 2022). Agricultural control measures such as planting resistant varieties, deep planting and irrigation have been applied to the early control of PTMs, but chemical control is still the main way to control PTMs in the process of potato production (Zheng et al., 2020). Due to the extensive use of chemical pesticides, the insect has developed varying degrees of resistance to organophosphorus, pyrethroids, and other pesticides (Yan et al., 2020). Therefore, in order to reduce the use of chemical pesticides and delay the development of resistance, it is urgent to develop efficient and safe green control technology for PTM (Yuan et al., 2017; Yuan et al., 2018; Yan et al., 2021).

Insect gut is an important organ connecting the host and the external environment. There are a large number of microorganisms involved in the life activities of the host (Jing et al., 2020). Insect gut microbes contain a large number of bacteria, archaea, fungi, and various protozoa (Gressel, 2018). Most of the microorganisms in the intestinal tract of insects are bacteria, mainly Actinobacteria, Firmicutes, Proteobacteria, and Bacteroidetes (Douglas, 2015; Sontowski and van Dam, 2020; Kucuk, 2020). The study found that the symbiotic bacterium *Candidatus Tremblaya phenacola* was stable throughout the life cycle of female individuals of *Phenacoccus solenopsis*, and its activity increased during oviposition, but decreased significantly at the late stage of male pupation. Genome sequencing and metabolic pathway analysis showed that the endosymbiont supported the growth and reproduction of the host by providing essential amino acids (Bai et al., 2024). There are significant structural differences in the intestinal microbial communities of nurse bees and forage bees in *Apis mellifera*, intestinal microorganisms can participate in the division of labor of social insects by affecting the behavioral phenotype of the host (Vernier et al., 2024). Intestinal microorganisms affect the aggressive behavior of *Drosophila* by regulating the synthesis and expression of octopamine (Jia et al., 2021). The gene expression between *Sitophilus oryzae* and its endosymbiont *Sodalis pierantonius* has a synergistic effect, including immunity, metabolism, metal control, apoptosis and bacterial stress response, which are intertwined and regulated by each other (Ferrarini et al., 2023). In addition, the composition and diversity of insect intestinal flora are also affected by many factors. The study indicated that the symbiotic bacterial community of herbivorous insects is affected by both the host insects and environment, and the host insects mainly determines the dominant bacteria, while the environment generally affects the secondary bacteria (Yang et al., 2023). *Diutina rugosa* and *Komagataeibacter saccharivorans* were significantly increased in the outbred population of *Bactrocera dorsalis*, and the changes of these floras were related to the changes of amino acid metabolism, which affected the physiological characteristics of flies (Wang et al., 2023). There were significant differences in the gut microbiome of female adults of *Colaphellus bowringi* in the diapause and non-diapause states (Liu et al., 2016). Furthermore, variations in host plant species, geographical regions, seasonal fluctuations, and population density can significantly influence the composition and diversity of intestinal microbiota (Martínez-Solís et al., 2020; Mogouong et al., 2020; Higuita Palacio et al., 2021; Yang et al., 2022).

At present, the research on the physiological and ecological functions of microorganisms mainly depends on traditional isolation and culture techniques. However, due to the complex living environment of microorganisms, *in vitro* culture cannot well simulate their complex symbiotic environment. Therefore, only a small number of microorganisms can be cultured in nature, and most of them cannot be cultured (Wang et al., 2020). With the development of molecular technology, DNA sequencing technology continues to innovate. The third-generation sequencing technology is a new type of sequencing technology that combines the advantages of high throughput, fast speed, long read length and low cost. Its biggest feature is that it does not require PCR amplification and can directly read the target sequence. Therefore, the false positive rate is greatly reduced, and the occurrence of common PCR errors such as base substitution and bias is avoided (Searle et al., 2023). Therefore, in this study, the composition and diversity of intestinal bacteria in different developmental stages of potato tuber moth was evaluated, aiming to clarify the key strains that have significant changes in the whole development cycle of potato tuber moth, and lay a foundation for the subsequent excavation of the physiological and ecological functions of key strains and the development and utilization of microbial resources.

## 2 Materials and methods

### 2.1 Insect rearing and sample collection

The PTMs was collected from a potato field in Banqiao Town, Xuanwei City, Yunnan Province (N26°05’52.3”, E104°04’27.5”), and a stable population was established by indoor breeding in the Insect Laboratory of the College of Plant Protection, Yunnan Agricultural University (Wang et al., 2022). A total of 200 eggs, 200 the 1st instar larvae, 100 the 2nd instar larvae, 50 the 3rd–4th instar larvae, 50 the male and female pupae, and 50 the male and female adults were selected, rinsed in sterile water for 1 min, transferred into 75% ethanol solution for disinfection for 30 s, and then rinsed twice in sterile water for 1 min each time. All operations were performed in an ultra-clean bench. After surface disinfection, eggs and 1st–2nd instar larvae were directly placed in a centrifuge tube containing 0.2 mL PBS buffer for grinding. The 3rd–4th instar larvae and male and female pupae were dissected in an ultra-clean bench, and the dissected intestine was placed in a centrifuge tube containing 0.2 mL PBS buffer for grinding. The male and female adults were dissected under the microscope, and the alcohol lamp was lit next to the microscope. The dissected intestine was placed in a centrifuge tube containing 0.2 mL PBS buffer for grinding.

### 2.2 DNA extraction and library sequencing

The total DNA of intestinal microorganisms was extracted according to the instructions of QIAamp DNA Stool Mini kit. DNA samples were extracted and tested for qualified backup. The egg samples were numbered E, the 1st–4th instar larvae samples were numbered A–D, the male and female pupa samples were numbered PF and PM, and the male and female adult samples were numbered AF and AM. Each sample had three replicates. After extracting the total DNA of the sample, the specific primers with Barcode were synthesized according to the full-length primer sequence, and the PCR amplification was performed. The products were purified, quantified and homogenized to form a sequencing library (SMRT Bell) (Ling et al., 2023). The constructed library was first subjected to library quality inspection, and the qualified library was sequenced by PacBio Sequel II.

### 2.3 Bioinformatics analysis

The data of PacBio Sequel II is in bam format. The CCS file is exported by smrtlink analysis software, and the data of different samples are identified according to the Barcode sequence and converted into fastq format data. After exporting PacBio offline data to CCS files, lima v1.7.0 software is used to identify CCS through barcode, and the obtained Raw-CCS sequence data is Barcode identified with CCS sequence to obtain Raw-CCS sequence data. Then cutadapt 1.9.1 software was used to identify and remove the primer sequence and filter the length to obtain the Clean-CCS sequence without the primer sequence. Finally, the dada2 in QIIME2 2020.6 software is used to denoise and remove the chimera sequence to obtain the final valid data (Non-chimeric CCS). The α and β diversity indexes were verified by using the QIIME2 2020.6 software, and the dada2 method in the software was used to denoise the sequence to obtain ASVs. Using SILVA as the reference database, the naive Bayes classifier combined with the comparison method was used to classify the feature sequences, and the species classification information corresponding to each feature could be obtained. Then, the community composition of each sample was counted at each level, and the QIIME software was used to generate the species abundance table at different classification levels, and then the R language tool was used to draw the community structure diagram at each taxonomic level of the sample (Xu et al., 2022). The 16S rRNA gene sequence was predicted in the KEGG functional database by PICRUSt2, and the correlation between metabolic pathways and strain abundance was analyzed by Spearman method (Chen et al., 2023).

### 2.4 Effects of strains feeding on the growth and development of PTM

We established five treatments: CK, AT, EM, SR, and EL, using normal leaf CK group and antibiotic-treated AT group as controls, to measure the growth and development indicators of potato tuber moth. In the CK group, larvae were fed untreated fresh leaves until pupation. The AT group larvae were fed untreated fresh leaves until the 2nd instar, then provided with antibiotic-treated leaves (immersed in a mixed antibiotic solution) for 48 h before resuming untreated fresh leaves until pupation. The EM, SR, and EL groups followed the same initial antibiotic treatment as AT group, but subsequently received leaves treated with *Enterococcus mundtii*, *Serratia rubidaea*, and *Enterobacter ludwigii* suspensions, respectively until pupation. Bacterial suspension preparation: Strains were isolated and purified from potato tuber moth intestines in previous experiments. Each strain was inoculated into 200 mL LB medium, cultured overnight at 180 rpm and 25°C, then standardized to an OD_600n*m*_ of 1. Antibiotic treatment: Fresh potato leaves were immersed in a mixed solution containing 500 μg/mL cefoperazone, 500 μg/mL chloramphenicol, 500 μg/mL streptomycin sulfate, and 500 μg/mL ciprofloxacin for 30 min (Wang et al., 2022), air-dried, and stored in sterile petri dishes. Leaves were replaced every 2 days during feeding. Each treatment included 5 replicates with 20 larvae per replicate. The larval mortality, adult emergence rate, pupal weight, pupal duration, male and female adult duration were recorded.

### 2.5 Data analysis

All data analysis was performed using SPSS 27.0. Statistical analysis was performed on the basis of variance normality and homogeneity hypothesis. One-way ANOVA analysis of variance was used. LSD method was used for pairwise comparison between groups. *P* < 0.05 indicated that the difference was significant. *P* < 0.01 indicated that the difference was extremely significant. Plot results using Graphpad Prism 8.0.

## 3 Results

### 3.1 Bacterial communities in different stage of PTM

**FIGURE 1:**
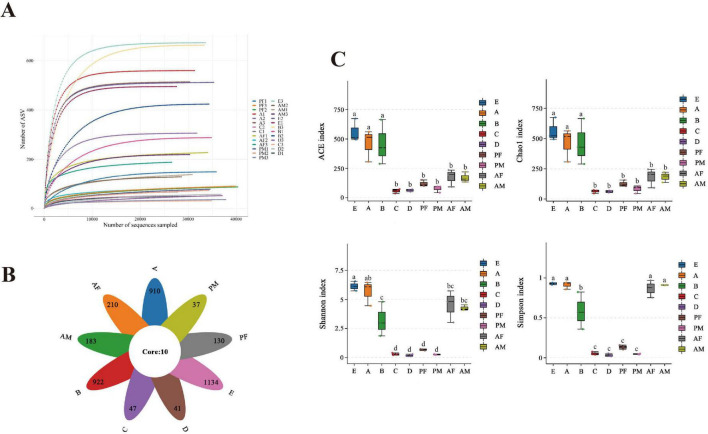
The diversity and comparison of the intestinal microbial community compositions in different PTM instars. **(A)** Rarefaction curve of bacterial diversity in different PTM instars. **(B)** Venn plots of common and unique bacterial species in the intestines of different PTM instars. **(C)** The diversity metrics ACE, Chao 1, Shannon, and the Simpson indexes of different PTM ages were estimated. Different lowercase letter marks above each group indicated that there were significant differences between different stages groups (one-way ANOVA, LSD *post-hoc* test, *P* < 0.05). Error bars represent ± SE of the mean. E, Egg; A, 1st instar; B, 2nd instar; C, 3rd instar; D, 4th instar; PF, Female pupae; PM, Male pupae; AF, Female adult; AM, Male adult.

Based on the PacBio sequencing platform, the intestinal bacteria of each instar of PTMs were sequenced by Single Molecule Real-Time Sequencing. A total of 9,34,669 Raw-CCSs were obtained from 27 samples, with an average of 34,617 Raw-CCSs per sample (Supplementary Table S1). After the original sequence was optimized by length filtering, denoising and chimera removal steps, a total of 9,07,198 valid sequences were obtained, with an effective sequence ratio of 97.06%, indicating that the sample has high accuracy. With the increase of the number of sequencing, the number of ASV gradually tended to be flat, indicating that the species in each sample would not increase significantly with the increase of the number of sequencing, and the sample sequence was sufficient for subsequent data analysis (Figure 1A). The division of ASV by the effective sequence showed that the number of microbiota in the egg was the highest, followed by the 1st and 2nd instar larvae, while the 3rd instar larvae, 4th instar larvae, and female pupae samples showed a considerable reduction (Figure 1B). Chao1 and ACE indexes (richness), Shannon and Simpson diversity indexes (evenness), showed that the diversity of 3rd instar larvae, 4th instar larvae, female pupae and male pupae samples was significantly lower than in the other stages (*p* < 0.05) (Figure 1C).

### 3.2 The composition and abundance of gut microbiota across the life cycle of PTM

Based on the SILVA database, 27 samples were combined and annotated to 31 phyla, 325 families, 714 genera, and 1,410 species (detailed information per sample in Supplementary Table S2). The PTM microbiota consists mainly of members of the phyla Proteobacteria and Firmicutes. The egg and the 1st instar larvae microbiota contains mostly Proteobacteria (50.56 and 53.02% on average) and in second place Firmicutes (38.03 and 38.05%). The 2nd- to the 4th-instar larvae and pupae microbiota showed similar phyla composition enriched, with the absolute dominance of Firmicutes (92.56%). Interestingly, the adult gut is dominated again by Proteobacteria (74.82%) followed by Firmicutes (23.20%). In addition, Bacteroidota and Actinobacteria were distributed in all ages, and the average relative abundance was between 0.03 ∼ 3.33% and 0.01 ∼ 1.09% (Supplementary Figure S1).

**FIGURE 2:**
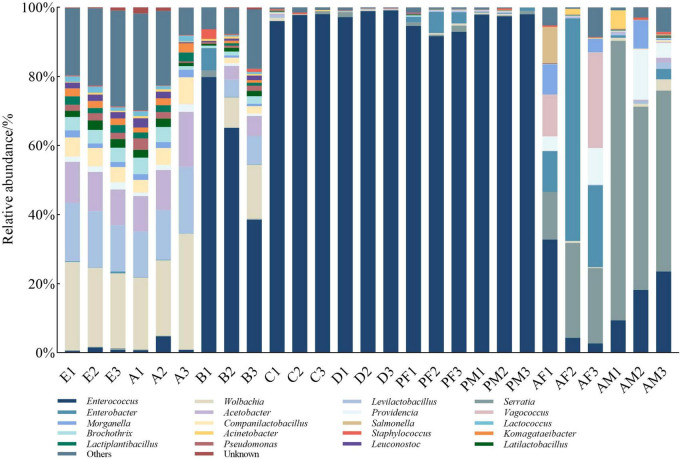
The top 20 of the relative abundance of microbial composition at the level of PTM. Samples representing different developmental stages of the entire life cycle are listed, and different colors indicate annotations for different bacterial genera. E, Egg; A, 1st instar; B, 2nd instar; C, 3rd instar; D, 4th instar; PF, Female pupae; PM, Male pupae; AF, Female adult; AM, Male adult.

When we examined lower taxonomic levels (Figure 2), we found that the most abundant genera in eggs and the 1st instar larvae were *Wolbachia* (23.41 and 25.4%), *Levilactobacillus* (15.5 and 15.64%), and *Acetobacter* (11.21 and 12.62%), all of them are Proteobacteria and Firmicutes. In 2nd instar larvae guts, two genera, *Enterococcus* (Firmicutes) were the most abundant (61.13%), followed by *Wolbachia* (8.15%). Subsequently, the abundance of *Enterococcus* was absolutely dominant during the period from the 3rd instar larvae to the male and female pupae (96.63% on average). *Serratia* has a high abundance in adults, especially in males (62.06%). Interestingly, we found that the abundance of *Enterobacter* was the highest in female adults (33.32%), followed by *Serratia* (21.07%), and showed different characteristics between adults. The overall difference in bacterial composition between stages was evident. However, older larvae and pupae showed greater similarity in contrast to other stages.

**FIGURE 3:**
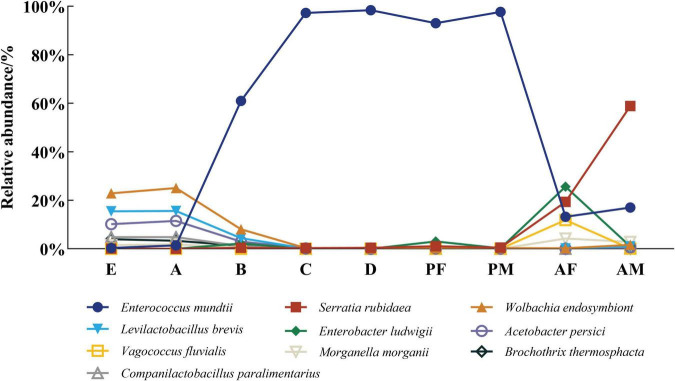
The dynamic changes of core flora in the life cycle of PTM at the species level. Columns represent the various developmental stages of the entire life cycle, and different colors represent the corresponding strains. E, Egg; A, 1st instar; B, 2nd instar; C, 3rd instar; D, 4th instar; PF, Female pupae; PM, Male pupae; AF, Female adult; AM, Male adult.

We found significant changes in the abundance of some intestinal commensal bacteria during the life cycle of the PTM at the species level (Figure 3). The relative abundance of *Enterococcus mundtii* was extremely low in eggs and the 1st instar larvae, higher in the 2nd instar larvae, and reached an extremely high level in the 3rd instar larvae, 4th instar larvae and pupae, exceeding 90%, before declining in the adult stage, while *Morganella morganii* showed an opposite pattern. We also discovered that *Companilactobacillus paralimentarius*, *Levilactobacillus brevis*, *Wolbachia endosymbiont*, *Acetobacter persici*, *Brochothrix thermosphacta* were present in high abundance exclusively in eggs and first instar larvae, and then gradually decreased to a very lower level. In particular, the abundance of *Serratia rubidaea* was higher only in the adult stage, and the abundance of *Enterobacter ludwigii* was higher only in the female adult.

**FIGURE 4:**
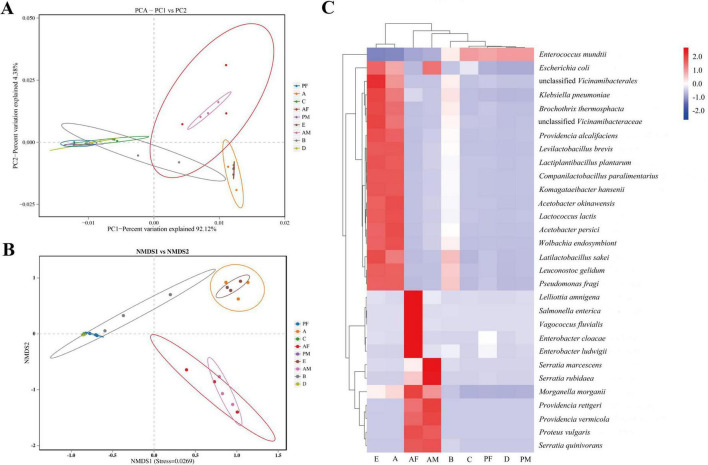
Gut microorganism dynamics across the life cycle of PTM. **(A)** Two-dimensional PCA of microbial communities using the Bray-Curtis distance calculation to measure different stage samples. Each point in the figure represents a sample, different colors represent different samples, the elliptical ring represents it as a 95% confidence ellipse. The abscissa represents the first principal component, and the percentage represents the contribution value of the first principal component to the sample difference. The ordinate represents the second principal component, and the percentage represents the contribution value of the second principal component to the sample difference. **(B)** NMDS based on the gut microbiota composition of PTM. Each point in the figure represents a sample, different colors represent different groups; the elliptical circle indicates that it is a 95% confidence ellipse, and the distance between points indicates the degree of difference. The where samples closer to each other on the coordinate plot exhibit higher similarity. **(C)** The heat map of the top 30 species in relative abundance at the species level. Each column represents the different developmental stages of the whole life cycle, and the vertical is the species classification group, which is clustered according to the similarity of the microbial abundance spectrum. The clustering tree on the left side is the species clustering tree, and the clustering tree above is the sample clustering tree, which reflects the similarity of community composition between samples. The value corresponding to the heat map is the *Z*-score standardization of the same species using the *R* scale function between different samples, and the color gradient from blue to red indicates the abundance from low to high between samples. E, Egg; A, 1st instar; B, 2nd instar; C, 3rd instar; D, 4th instar; PF, Female pupae; PM, Male pupae; AF, Female adult; AM, Male adult.

We performed non-metricMulti-dimensional scaling (NMDS) analysis and principal component (PCA) analysis based on weighted-unifracdissimilarity to analyze the differences in the microbiota composition between the nine groups (egg, 1st- to the 4th-instar larvae, male pupa, female pupa, male adult, and female adult) of samples. The analysis showed that the samples were grouped according to their stage, indicating that the PTM gut microbiota is stage specific. Both 3th- to the 4th-instar larvae and pupa cluster broadly overlapped. The egg and 1st instar larvae groups were closely related, and the male adult and female adult were closely related. In the PCA analysis, PC1 accounted for 92.12% of the total variance, and PC2 accounted for 4.38% (Figure 4A). In the NMDS analysis, stress = 0.0269, which indicated that the grouping and sampling was reliable (Figure 4B). Heat maps of the top 30 microbes in relative abundance at the genus level were drawn (Figure 4C). The microbiological structures of eggs and 1st instar larvae were similar, and those of the female adult and male adult were similar. *E. mundtii* was the only species with high abundance in the 3rd instar larvae, 4th instar larvae and pupae, whereas the abundance of other bacteria was very low. However, the dynamics of the structure of these four similar groups were completely the opposite.

### 3.3 Microbial interaction network in PTM

**FIGURE 5:**
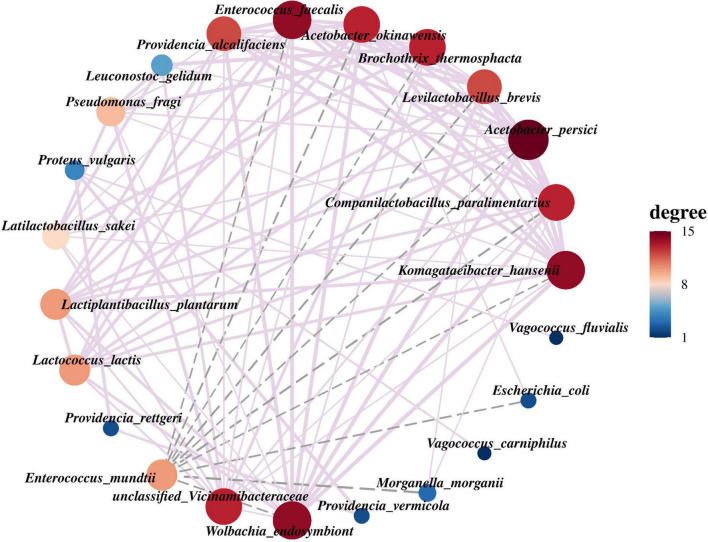
The interaction network among the top 30 bacterial species in the relative abundance of PTM. The node represents the group at the species level, the connection between each two nodes represents the correlation between the classification unit pairs, the thickness of the connection represents the strength of the correlation, and the size of the node represents the number of related objects. The purple solid line indicates positive correlation, and the gray dotted line indicates negative correlation.

According to the abundance and variation of bacterial species in PTM samples, Spearman rank correlation analysis was performed and the correlation network was constructed by screening data (*r* > 0.1, *p* < 0.05). In the network, *A. persici* exhibits the highest connectivity with other bacteria. Other strains demonstrating substantial associations include *W. endosymbiont*, *Komagataeibacter hansenii*, *Enterococcus faecalis*, *Acetobacter okinawensis*, *B. thermosphacta*, and *C. paralimentarius*, which frequently demonstrate co-occurrence patterns with multiple bacterial strains. In contrast, our analysis revealed that *E. mundtii* did not exhibit positive correlations and formed significant negative correlations with multiple bacterial strains, including *E. faecalis*, *A. okinawensis*, *B. thermosphacta*, *Levilactobacillus brevis*, *A. persici*, *C. paralimentarius*, *K. hansenii*, *Escherichia coli*, *M. morganii* and *W. endosymbiont* (Figure 5).

### 3.4 *Enterococcus mundtii* enhanced the carbohydrate metabolism of PTM

**FIGURE 6:**
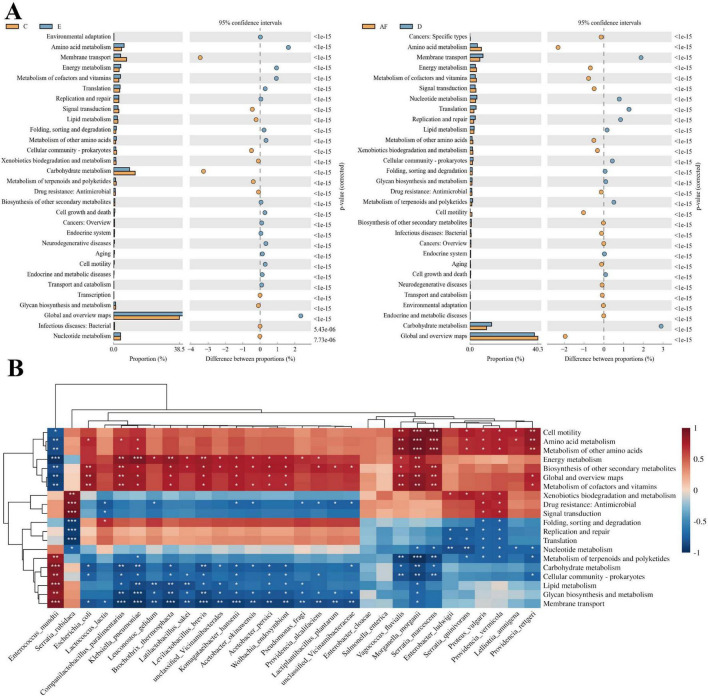
KEGG function prediction of PTM intestinal bacteria. **(A)** The difference analysis diagram of KEGG metabolic pathways between eggs and 3rd instar larvae, 4th instar larvae and female adults at the second level was analyzed. Different colors in the diagram represent different groups. The left diagram shows the abundance ratio of different functions in two samples or two groups of samples, the middle is the difference ratio of functional abundance in the 95% confidence interval, and the rightmost value is *p*-value. **(B)** Heatmap of correlation between high-abundance bacterial species and metabolic pathways at different developmental stages of PTM (Spearman’s method, ***P < 0.001; **0.001 ≤ *P* < 0.01; *0.01 ≤ *P* < 0.05).

The PICRUSt2 function of the top 30 strains were predicted with relative abundance across the whole life cycle. The results showed that the relative abundance of different strains in each functional category was slightly different at the second classification level, but the functional categories with the highest relative abundance of all strains were global and overview maps, and the relative abundance was ranged from 36.33 to 45.10%. The second is carbohydrate metabolism, and the relative abundance is ranged from 7.54 to 12.32%. Among them, the lowest was observed in *Pseudomonas fragi*, while the highest was found in *E. mundtii* (Supplementary Figure S2). The differences of KEGG metabolic pathways in intestinal symbiotic bacteria communities of eggs and the 3rd instar larvae, female adults and 4th instar larvae were analyzed. We found that the global and overview map and amino acid metabolism of the symbiotic bacteria community in the egg were significantly stronger than those of the intestinal symbiotic bacteria community of the 3rd instar larvae, while the membrane transport function and carbohydrate metabolism of the intestinal symbiotic bacteria community of the 3rd instar larvae were significantly stronger than those of the symbiotic bacteria community in the egg. The carbohydrate metabolism and membrane transport of the intestinal symbiotic bacteria community of the 4th instar larvae were significantly stronger than those of the intestinal symbiotic bacteria community in the female adults, while the global and overview map function and amino acid metabolism of the intestinal symbiotic bacteria community of the female adults were significantly stronger than those of the intestinal symbiotic bacteria community of the 4th instar larvae (Figure 6A). Therefore, the intestinal symbiotic bacteria in the 3rd and 4th instar larvae have strong membrane transport and carbohydrate metabolism.

We conducted Spearman’s rank correlation analysis between the relative abundances of the top 30 bacterial strains and metabolic pathways. The results revealed that *E. mundtii*, as a predominant core species, exhibited significant positive correlations with six metabolic categories: metabolism of terpenoids and polyketides (*r* = 0.828, *P* < 0.01), carbohydrate metabolism (*r* = 0.917, *P* < 0.001), cellular community-prokaryotes (*r* = 0.879, *P* < 0.01), lipid metabolism (*r* = 0.916, *P* < 0.001), glycan biosynthesis and metabolism (*r* = 0.840, *P* < 0.01), and membrane transport systems (*r* = 0.933, *P* < 0.001). Notably, inverse correlations were observed between *E. mundtii* abundance and seven other functional categories: cell motility (*r* = –0.783, *P* < 0.05), amino acid metabolism (*r* = –0.850, *P* < 0.01), metabolism of other amino acids (*r* = –0.840, *P* < 0.01), energy metabolism (*r* = –0.962, *P* < 0.001), biosynthesis of other secondary metabolites (*r* = –0.843, *P* < 0.01), global and overview maps (*r* = –0.900, *P* < 0.01), and metabolism of cofactors and vitamins (*r* = –0.895, *P* < 0.01) (Figure 6B).

### 3.5 Gut microbiomes facilitates the growth and development of PTM

**FIGURE 7:**
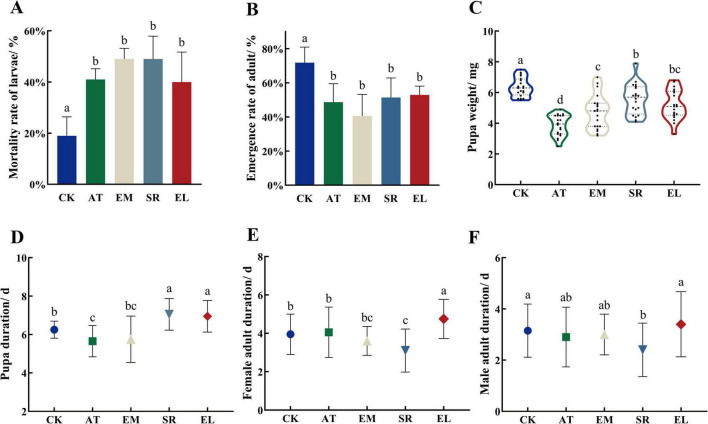
The influences of EM, SR, and EL on the growth and development of PTM. Different lowercase letter marks above each group indicated that there were significant differences between different stages groups (one-way ANOVA, LSD *post-hoc* test, *P* < 0.05). Error bars represent ± SE of the mean. **(A)** Mortality rate of PTM larvae inoculated with different bacterial strains. **(B)** Emergence rate of PTM adult inoculated with different bacterial strains. **(C)** Pupa weight of PTM inoculated with different bacterial strains. **(D)** Pupa duration of PTM inoculated with different bacterial strains. **(E)** Female adult duration of PTM inoculated with different bacterial strains. **(F)** Male adult duration of PTM inoculated with different bacterial strains.

According to the sequencing results, *E. mundtii*, *E. ludwigii*, and *S. rubidaea* were the dominant bacteria, so we evaluated the function of *E. mundtii*, *E. ludwigii*, and *S. rubidaea* on the growth and development of PTM after feed PTM with these strains. Compared with the antibiotic treatment group, the mortality rate of the larvae was significantly higher than that of the control group (*P* < 0.01), but there was no significant difference between the treatments and the refeeding strains (Figure 7A). Similarly, the same as in the adult emergence rate, and the antibiotic treatment group and the refeeding strain group were significantly lower than the control group (*P* < 0.05), it indicated that antibiotic treatment had a great influence on the growth of larvae and adult emergence (Figure 7B).

The pupa weight of PTM after the inoculation of antibiotic, *E. mundtii*, *S. rubidaea*, *E. ludwigii* was 3.87 ± 0.15, 4.75 ± 0.25, 5.56 ± 0.99, and 5.19 ± 0.95 mg, they were all less than that in CK (6.36 ± 0.14 mg). Most importantly, the pupa weight of PTM after the inoculation of EM, SR and EL was significantly greater than that in the antibiotic treatment group (*P* < 0.05) (Figure 7C). The pupa period was 5.65 ± 0.18, 5.75 ± 0.27, 7.05 ± 0.18, 6.95 ± 0.18, and 6.25 ± 0.10 d under the treatment of antibiotic, *E. mundtii*, *S. rubidaea*, *E. ludwigii*, and CK, respectively. So the SR and EL groups could also significantly prolong the pupa period (*P* < 0.05) (Figure 7D).

The female adult duration of each experimental group is shown in Figure 7E, it was 4.05 ± 0.29, 3.60 ± 0.17, 3.10 ± 0.25, 4.75 ± 0.23, and 3.95 ± 0.23 d under the treatment of antibiotic, *E. mundtii*, *S. rubidaea*, *E. ludwigii* and CK, respectively. The EL group was significantly higher than that in the control group and antibiotic treatment group, while the SR group was significantly lower than that in the control group and antibiotic treatment group (*P* < 0.01). The male adult duration of each experimental group is shown in Figure 7F, it was 2.90 ± 0.26, 3.00 ± 0.18, 2.40 ± 0.23, 3.40 ± 0.28, and 3.15 ± 0.23 d, under the treatment of antibiotic, *E. mundtii*, *S. rubidaea*, *E. ludwigii*, and CK, respectively. The results showed that the duration of male adults was significantly shortened after the feeding with *S. rubidaea* (*P* < 0.05). There were no significant difference between the EM groups and the control group, indicating that the bacteria did not significantly affect the development of pupa and adult.

## 4 Discussion

In this study, single-molecule real-time sequencing technology (16S rRNA gene sequencing) was used to analyze the microbial communities at different stages (egg, larva, pupa, and adult) of the life cycle of PTM. Alpha diversity analysis showed that the richness and diversity of eggs were the highest in all developmental stages, and then the diversity index decreased with the growth and development. The richness and diversity reached the lowest at the 4th instar, while the richness and diversity of adults were second only to eggs and 1st instar larvae. The microbial diversity was the highest in the egg stage of *Spodoptera frugiperda*, and the microbial diversity decreased sharply after the eggs were hatched into larvae. The microbial diversity of the 6th instar larvae was the highest in the larval stage, and the community richness was the lowest in the adult stage (Li et al., 2022). The intestinal bacterial diversity of *Brithys crini* adults was significantly lower than that of eggs and larvae (González-Serrano et al., 2020). In the study of the diversity of intestinal bacteria in different developmental stages of *Helicoverpa armigera*, it was found that the richness and diversity of intestinal bacteria in the 1st instar larvae and adults were the highest, and the richness and diversity of the 4th instar larvae were the lowest (Zhao et al., 2023). Based on the above results, we found that there were differences in the diversity and richness of intestinal bacteria in eggs, larvae, pupae, and adults of different lepidopteran insects. In all developmental stages, the egg stage usually has high richness and diversity, which may be related to the vertical transmission of symbiotic bacteria from the mother to the next generation to ensure that the newly hatched larvae can quickly adapt to the environment and host plants. Some adults have a long duration, and after a long period of feeding and contact with the external environment, the symbiotic bacteria in the body have higher richness and diversity, while the adults of PTM have a short duration and die soon after reproduction, and the food of laboratory reproduction population is single, which may be the reason for the low diversity and richness of intestinal symbiotic bacteria in adults.

Our study found that the intestinal bacteria of PTM are mainly composed of Firmicutes and Proteobacteria. The study of Lepidoptera insects by modern molecular techniques showed that the intestinal bacteria of Lepidoptera insects mainly included Proteobacteria (56.98%) and Firmicutes (22.15%) (Shao et al., 2024). Paniagua Voirol et al. (2018). Compared the gut microbiome of 30 lepidopteran insects and found that *Enterococcus* and *Enterobacter* were widely present in lepidopteran insects. At the species level, we found significant changes in the abundance of nine bacteria during the life cycle of the potato tuber moth. Among them, *C. paralimentarius*, *M. morganii*, *W. endosymbion*, *A. persici*, and *B. thermosphacta* had higher abundance in eggs and 1st instar larvae. *Wolbachia* is a bacterium that is transmitted vertically from mother to offspring within cells, and it is widely present in arthropods and can enhance the host’s detoxification ability, increase the host’s resistance to drugs, and have antiviral effects, as well as affecting the host’s gut microbiome (Grobler et al., 2018; Liu and Guo, 2019; Pimentel et al., 2021; Ourry et al., 2021; Ye et al., 2024). *Lactobacillus* has been discovered to inhibit ecdysone hormone signaling, delay pupation, and detoxify (Daisley et al., 2018; Xiong et al., 2024). *Acetobacter pomorum* can delay the development of *Drosophila suzukii* larvae and reduce the body weight of eclosion adults, induce larval immune response and down-regulate genes involved in digestion and juvenile hormone metabolism (Bing et al., 2024). *Lactococcus*, *Enterobacter* and *Klebsiella* were added to the feed of Mediterranean fruit flies larvae and adults, and it was found that probiotic clusters could significantly improve the viability, flight ability and mating competitiveness of Mediterranean fruit flies (Haytham et al., 2024). Therefore, we speculate that the dominant bacteria in the early growth and development of PTM are mainly the help group host to quickly adapt to the environment and have the effect of resisting pathogens. Following this phase, the relative abundance of *E. mundtii* increased rapidly and became absolutely dominant, while the previously dominant bacterial species exhibited a sharp decline to near-zero levels. Li et al. (2023). Showed that the expression of antimicrobial peptides (AMPs) could be activated by *E. mundtii* in the intestinal tract of *Hyphantria cunea* larvae, and AMPs have broad-spectrum antibacterial activity, inhibiting other bacterial competitors and regulating the number of endosymbionts (Sta̧czek et al., 2023). Therefore, we speculate that *E. mundtii* may inhibit the early dominant species such as *Wolbachia* by secreting AMPs and quickly occupy the niche. *Enterobacter* can shorten the developmental period of Drosophila larvae, reduce the mortality rate of larval stage, increase pupal weight and improve fecundity (Kyritsis et al., 2019). *Serratia* can increase the body weight and body size of aphids, reduce the developmental duration of nymphs, improve the fecundity of adult aphids, and shorten the longevity of aphids, indicating that pea aphids carrying *Serratia* will strengthen the ecological fitness of the population at the expense of longevity (Wang et al., 2024). In this study, it was found that the abundance of *Enterobacter* and *Serratia* increased significantly in the adult stage, which was speculated to be related to the two strains of bacteria assisting the host to reproduce. There were significant differences in the intestinal microbial diversity of *Apolygus lucorum* at different life stages, and especially in the adult stage, the relative abundance of Serratia increased significantly, which was consistent with the results of this study (Guo et al., 2024).

Many studies have shown that *Enterococcus mundtii* plays a variety of important functions in the host. In the intestinal tract of *Spodoptera litura* larvae, *E. mundtii* can stably colonize in time and space and has a rich lysine synthesis pathway in the hindgut, which helps the host to grow and develop (Mazumdar et al., 2021). *Enterococcus* isolated from the intestinal tract of *Plutella xylostella* enhance resistance to chlorpyrifos by affecting the host immune system (Xia et al., 2018). *E. mundtii* in the gut of *Tuta absoluta* has detoxification effect on chlorantraniliprole (Chen et al., 2024). Antimicrobial peptides in the gut of *Galleria mellonella* can kill *Enterococcus* in the larval gut, and the removal of *Enterococcus* can accelerate pupation and drive insect metamorphosis (Kong et al., 2023). The *Enterococcus* dominated bacterial community in the gut of *Hyles euphorbiae* plays an important role in the host’s tolerance to feeding on poisonous plants (Vilanova et al., 2016). The dominant symbiotic bacteria of *Spodoptera littoralis* a is *E. mundtii*, and it can secrete stable bacteriocins to resist invasive bacteria, but it does not resist other intestinal symbiotic bacteria and promotes the normal development of the host intestinal microflora, even if the niche in the intestine is blocked in the early stage, *E. mundtii* can effectively overcome the invasive bacteria and rapidly reproduce, colonize in the digestive tract, and stably maintain throughout the life cycle of the larvae (Shao et al., 2017). In this study, we found that the abundance of *E. mundtii* was extremely high in the 3rd and 4th instar larvae, as well was in pupae, low in the early and late stages of development, and had high carbohydrate metabolism ability. This further proves that *E. mundtii* can help larvae to carry out nutritional metabolism, and may also regulate insect metamorphosis.

## 5 Conclusion

The richness and diversity of intestinal microbiota in eggs were higher than that in other stages, and the lowest richness and diversity of intestinal microbiota was in the 4th instar larvae, while those in adults were second only to those in eggs and the 1st instar larvae. The dominant bacteria of PTM were different in different stages. *Wolbachia endosymbiont* was the dominant species in eggs and the 1st instar larvae, *Enterococcus mundtii* was the dominant strain in the 2nd–4th instar larvae and pupae, *Enterobacter ludwigii* and *Serratia rubidaea* were the dominant strains in female adults and male adults, respectively. In addition, the dynamics process of nine strains of bacteria in the whole life cycle of PTM was also clarified.

The gut-associated bacteria facilitate insect host growth and development. *E. mundtii* also has strong carbohydrate metabolism ability, which may help the host insect to digest and absorbe nutrients. Therefore, we re-fed bacteria after antibiotic treatment to observe their effects on the growth and development of PTM and found that changes in the intestinal flora of larvae will affect growth and development. Compared with the antibiotic treatment group, *E. mundtii*, *S. rubidaea*, and *E. ludwigii* could significantly increase pupal weight and pupal period. However, *S. rubidaea* significantly shortened the adult duration.

We can thus appreciate the inter-relationship between intestinal flora during the whole life cycle, the influence of the changes in intestinal microflora of larvae on the growth and development of insects, and the mechanism of action of such an influence. These results lay a foundation for the development and utilization of key microorganisms and provide a new direction for the green control of PTM.

## Author contribution

QF: Writing – original draft, Writing – review & editing. WW: Writing – original draft, Writing – review & editing. BC: Writing – original draft, Writing – review & editing. YH: Writing – original draft. RM: Writing – original draft. EZ: Writing – original draft. SJ: Writing – original draft. HC: Writing – original draft. GX: Writing – review & editing. GD: Writing – review & editing.

## Data Availability

The datasets presented in this study can be found in online repositories. The names of the repository/repositories and accession number(s) can be found at: https://www.ncbi.nlm.nih.gov/, PRJNA1268744.
