# Supplementary material for: Longitudinal dynamics of intestinal bacteria in the life cycle and their effects on growth and development of potato tuber moth
Source: Front Microbiol. 2025 Jun 18;16:1542589. doi: 10.3389/fmicb.2025.1542589 (PMC12216665; doi:10.3389/fmicb.2025.1542589)
Supplement: Supplementary file 1 [file Supplementary_file_1.docx]

Supplementary Material

Supplementary table 1 Sequencing data results of samples

| Samples | Raw CCS | Clean CCS | Denoised CCS | Non-chimeric CCS | Effective sequence ratio(%) |
| --- | --- | --- | --- | --- | --- |
| E1 | 28367 | 28355 | 28328 | 27865 | 98.23 |
| E2 | 36333 | 36315 | 36196 | 35759 | 98.42 |
| E3 | 36310 | 36301 | 36159 | 35413 | 97.53 |
| A1 | 35839 | 35817 | 35772 | 35443 | 98.90 |
| A2 | 31854 | 31828 | 31752 | 31359 | 98.45 |
| A3 | 32624 | 32601 | 32582 | 32180 | 98.64 |
| B1 | 35421 | 35419 | 35397 | 35096 | 99.08 |
| B2 | 35121 | 35119 | 35090 | 34737 | 98.91 |
| B3 | 34934 | 34931 | 34860 | 34128 | 97.69 |
| C1 | 28257 | 28244 | 28217 | 28213 | 99.84 |
| C2 | 28207 | 28189 | 28181 | 28176 | 99.89 |
| C3 | 35237 | 35219 | 35206 | 35150 | 99.75 |
| D1 | 32084 | 32067 | 32059 | 31984 | 99.69 |
| D2 | 37178 | 37177 | 37176 | 37169 | 99.98 |
| D3 | 37308 | 37306 | 37306 | 37295 | 99.97 |
| PF1 | 36699 | 36679 | 36652 | 36592 | 99.71 |
| PF2 | 40860 | 40855 | 40822 | 40799 | 99.85 |
| PF3 | 40632 | 40629 | 40625 | 40213 | 98.97 |
| PM1 | 35108 | 35106 | 35063 | 35045 | 99.82 |
| PM2 | 34766 | 34765 | 34754 | 34734 | 99.91 |
| PM3 | 38223 | 38221 | 38215 | 37886 | 99.12 |
| AF1 | 37224 | 37216 | 37179 | 34428 | 92.49 |
| AF2 | 31276 | 31252 | 31226 | 29448 | 94.16 |
| AF3 | 33786 | 33786 | 33315 | 26910 | 79.65 |
| AM1 | 34585 | 34580 | 34563 | 31337 | 90.61 |
| AM2 | 32880 | 32864 | 32843 | 28870 | 87.80 |
| AM3 | 33556 | 33528 | 33495 | 30969 | 92.29 |
| Total | 934669 | 934369 | 933033 | 907198 | 97.06 |

Supplementary table 2 Number of species at each classification level

| Sample | Phylum | Class | Order | Family | Genus | Species |
| --- | --- | --- | --- | --- | --- | --- |
| E1 | 20 | 36 | 86 | 139 | 225 | 297 |
| E2 | 22 | 40 | 86 | 138 | 220 | 313 |
| E3 | 23 | 44 | 106 | 168 | 280 | 382 |
| A1 | 23 | 44 | 91 | 154 | 226 | 311 |
| A2 | 17 | 34 | 76 | 131 | 212 | 298 |
| A3 | 21 | 30 | 69 | 110 | 164 | 224 |
| B1 | 17 | 32 | 59 | 92 | 131 | 186 |
| B2 | 19 | 37 | 78 | 130 | 209 | 298 |
| B3 | 23 | 41 | 95 | 156 | 270 | 403 |
| C1 | 7 | 11 | 18 | 28 | 43 | 62 |
| C2 | 5 | 6 | 13 | 23 | 33 | 45 |
| C3 | 4 | 5 | 9 | 17 | 20 | 24 |
| D1 | 5 | 6 | 14 | 24 | 36 | 45 |
| D2 | 7 | 9 | 20 | 31 | 41 | 52 |
| D3 | 6 | 8 | 17 | 29 | 44 | 50 |
| PF1 | 18 | 25 | 48 | 73 | 93 | 113 |
| PF2 | 9 | 13 | 24 | 35 | 54 | 69 |
| PF3 | 11 | 15 | 24 | 36 | 52 | 69 |
| PM1 | 7 | 10 | 22 | 34 | 52 | 65 |
| PM2 | 8 | 12 | 23 | 37 | 52 | 62 |
| PM3 | 4 | 5 | 9 | 15 | 17 | 20 |
| AF1 | 8 | 10 | 25 | 49 | 84 | 128 |
| AF2 | 7 | 10 | 21 | 29 | 45 | 62 |
| AF3 | 8 | 10 | 21 | 33 | 56 | 83 |
| AM1 | 15 | 20 | 36 | 55 | 78 | 107 |
| AM2 | 12 | 14 | 27 | 43 | 70 | 89 |
| AM3 | 13 | 19 | 45 | 75 | 114 | 156 |
| Total | 31 | 65 | 169 | 325 | 714 | 1410 |


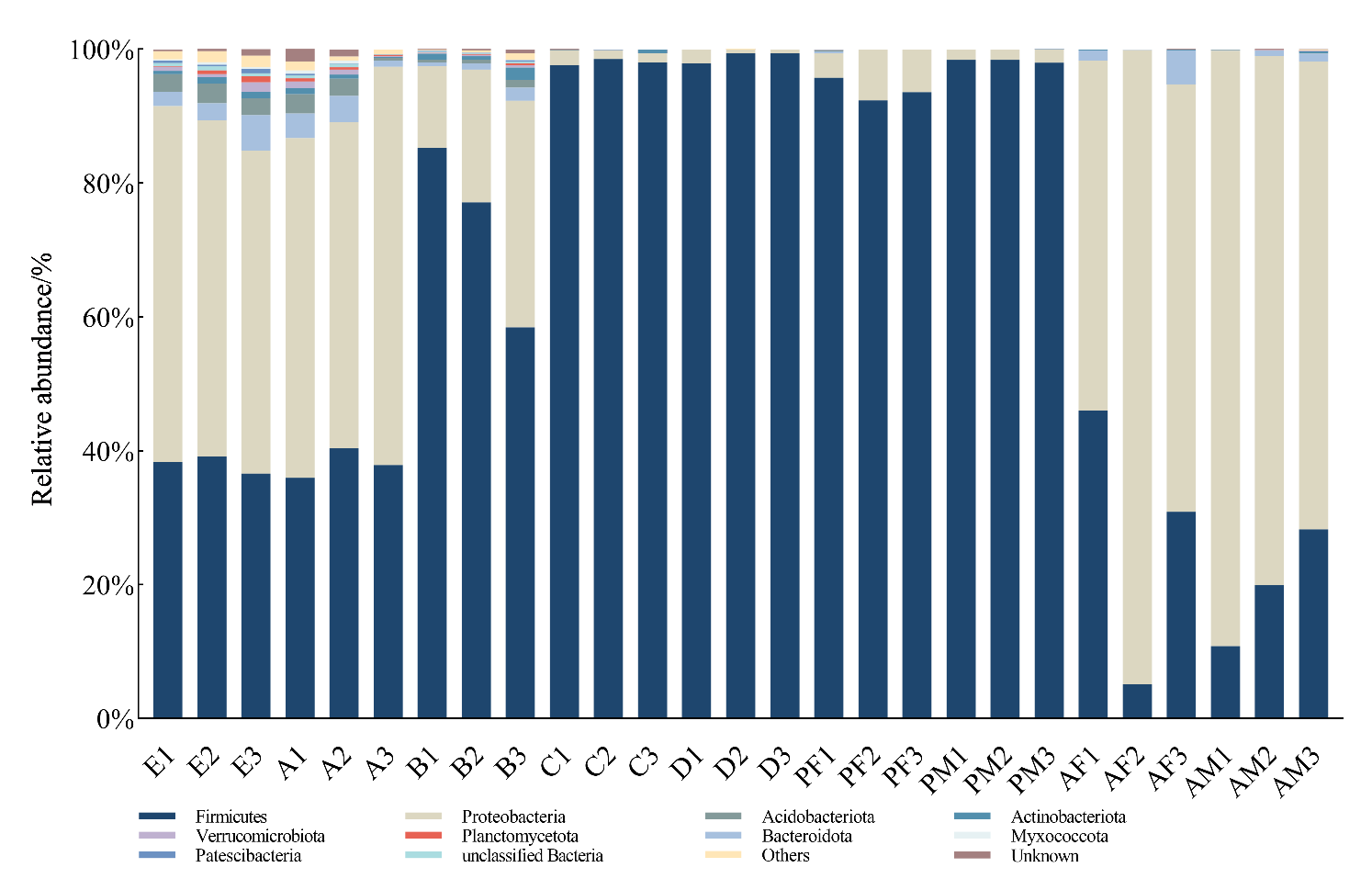


Supplementary Figure 1 The composition of gut samples in different developmental stages of PTM at phylum level


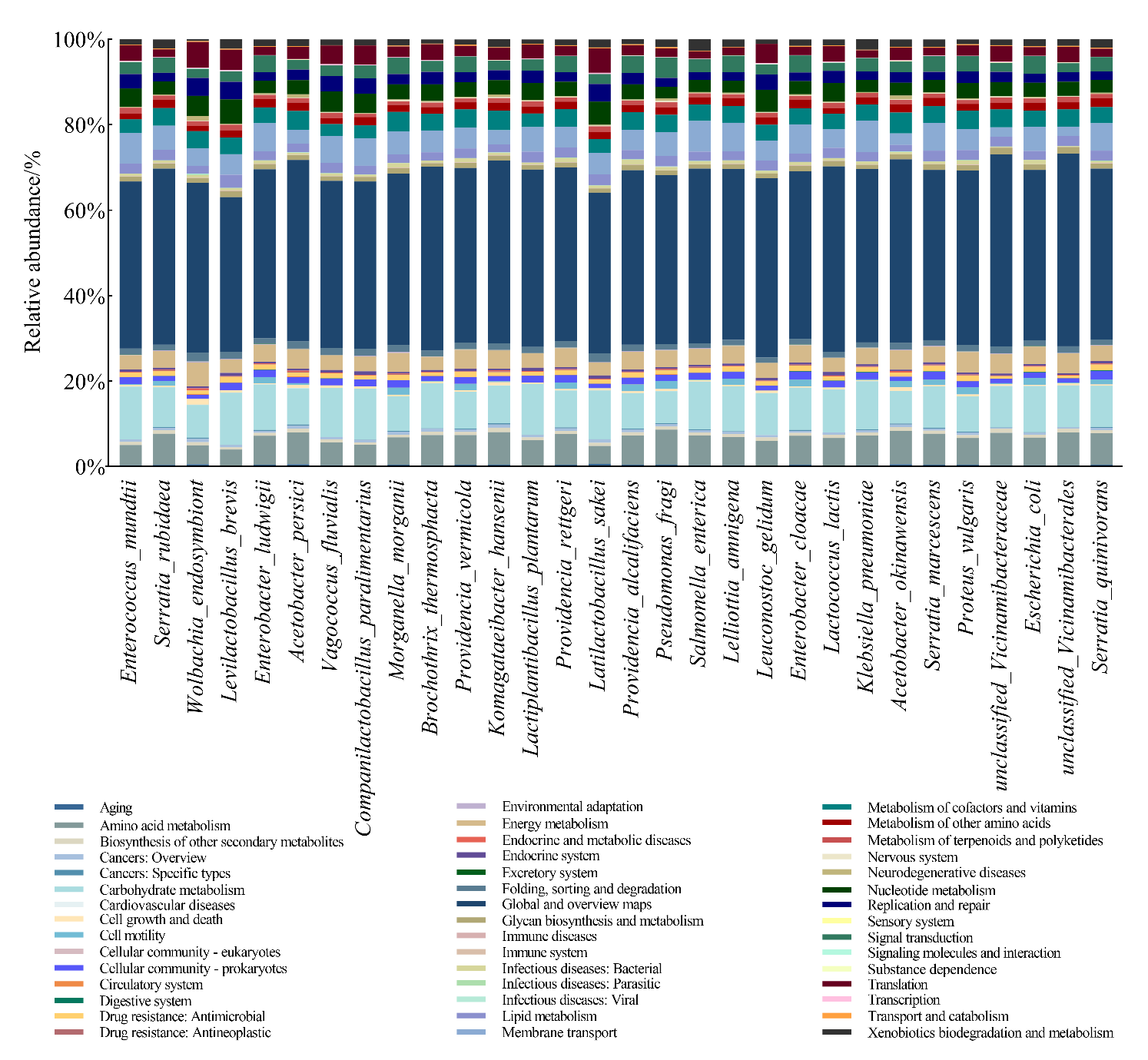


Supplementary Figure 2 The KEGG metabolic pathway histogram of the top 30 strains with relative abundance, the abscissa is the species, and the ordinate is the relative abundance percentage of the metabolic pathway.
